# Measuring “Waiting” Impulsivity in Substance Addictions and Binge Eating Disorder in a Novel Analogue of Rodent Serial Reaction Time Task

**DOI:** 10.1016/j.biopsych.2013.05.013

**Published:** 2014-01-15

**Authors:** Valerie Voon, Michael A. Irvine, Katherine Derbyshire, Yulia Worbe, Iris Lange, Sanja Abbott, Sharon Morein-Zamir, Robyn Dudley, Daniele Caprioli, Neil A. Harrison, Jonathan Wood, Jeffrey W. Dalley, Edward T. Bullmore, Jon E. Grant, Trevor W. Robbins

**Affiliations:** a Department of Psychiatry, University of Cambridge, Cambridge, United Kingdom; b Behavioural and Clinical Neuroscience Institute, University of Cambridge, Cambridge, United Kingdom; c Department of Psychology, University of Cambridge, United Kingdom; d Cambridgeshire and Peterborough NHS Foundation Trust, Cambridge, United Kingdom; e Brighton and Sussex Medical School, University of Sussex, Brighton, United Kingdom; f Department of Psychiatry and Behavioral Neuroscience, University of Chicago, Illinois

**Keywords:** Binge eating, impulsivity, motivation, obesity, premature responding, substance use disorders

## Abstract

**Background:**

Premature responding is a form of motor impulsivity that preclinical evidence has shown to predict compulsive drug seeking but has not yet been studied in humans. We developed a novel translation of the task, based on the rodent 5-choice serial reaction time task, testing premature responding in disorders of drug and natural food rewards.

**Methods:**

Abstinent alcohol- (*n* = 30) and methamphetamine-dependent (*n* = 23) subjects, recreational cannabis users (*n* = 30), and obese subjects with (*n* = 30) and without (*n* = 30) binge eating disorder (BED) were compared with matched healthy volunteers and tested on the premature responding task.

**Results:**

Compared with healthy volunteers, alcohol- and methamphetamine-dependent subjects and cannabis users showed greater premature responding with no differences observed in obese subjects with or without BED. Current smokers exhibited greater premature responding versus ex-smokers and nonsmokers. Alcohol-dependent subjects also had lower motivation for explicit monetary incentives. A Motivation Index correlated negatively with alcohol use and binge eating severity.

**Conclusions:**

Premature responding on a novel translation of a serial reaction time task was more evident in substance use disorders but not in obese subjects with or without BED. Lower motivation for monetary incentives linked alcohol use and binge eating severity. Our findings add to understanding the relationship between drug and natural food rewards.

Impulsivity can be broadly divided into decisional and motoric subtypes. Here we focus on a specific form of motor impulsivity, namely anticipatory or premature responding (1). In preclinical studies, premature responding is studied with the 5-choice serial reaction time task (5-CSRTT), a test for visual attention in which rodents monitor and respond to unpredictable visual targets (1). Premature responding is measured as anticipatory responding before target onset. Other forms of motor impulsivity include motor response inhibition or the inability to inhibit a prepotent motor response. The other major subgroup of impulsivity, decisional impulsivity, includes delay discounting—the tendency to select a smaller immediate reward over a larger delayed reward—and reflection impulsivity—the tendency to make rapid decisions without adequate consideration of options. These various subtypes of impulsivity are associated with broadly distinct but partially overlapping neural networks and neurochemical substrates 2, 3, 4.

Substance use disorders are commonly associated with high impulsivity [reviewed in Perry and Carroll (3)], which can occur both as a consequence of and a predictor of substance use disorders. In rodents, premature responding is elevated after methamphetamine (5) and alcohol withdrawal (6) and is also influenced by cannabinoid receptor CB1 receptor antagonists (7). High levels of premorbid premature responding and impulsive choice have also been shown to predict the transition to compulsive cocaine use in rodents (8), substantiating a potential role of premature responding as a predictor of future risk for substance use disorders. Although premature responding has been extensively studied in experimental animal models, premature responding with an analogous task has not yet been translated to studies in humans.

We developed a novel translation of the 5-CSRTT to assess premature responding in abstinent alcohol- and methamphetamine-dependent subjects and recreational cannabis users, compared with age- and gender-matched healthy volunteers. To compare drug versus natural rewards, we also assessed premature responding in obese subjects with and without binge eating disorder (BED). We assessed premature responding as a primary outcome and motivation for explicit reward as an exploratory measure. We hypothesized that premature responding would be elevated in abstinent alcohol- and stimulant-dependent subjects and recreational cannabis subjects. We hypothesized that obese subjects with BED would be elevated in premature responding, similarly to those with substance addiction.

## Methods and Materials

### Recruitment

Abstinent subjects with alcohol dependence (EtOH) (*n* = 30), obese subjects (>30 body mass index [BMI]) with BED (*n* = 30), obese control subjects without BED (*n* = 30), and recreational cannabis users (Cann) (*n* = 30) were recruited via community and university-based advertisements in Cambridge. Age- and gender-matched healthy volunteers (HV) (1:1 HV matching were used for EtOH, BED, and obese control subjects; 1:2 HV matching was used for Cann) were recruited via community- and university-based advertisements in Cambridge (HV: *n* = 30, *n* = 30, *n* = 30, *n* = 60, respectively). A total of 110 HV were recruited in Cambridge. Abstinent methamphetamine-dependent subjects (Meth) (*n* = 23) were also recruited from an inpatient rehabilitation center in Eden Prairie, Minnesota. Twenty age-matched HV were recruited from community advertisements in Minneapolis. Primary diagnoses were confirmed by a psychiatrist with the DSM IV-TR criteria for substance dependence or Research Diagnostic Criteria for BED (9). None of the Cann subjects fulfilled criteria for dependence.

Subjects >18 years old were included. The HV, EtOH, obese BED, obese control subjects, and Cann subjects were excluded if they had a current major depression or other major psychiatric disorder including substance addiction (except nicotine), major medical illness, or were taking psychotropic medications. The EtOH subjects were tested 2 weeks–1 year after abstinence and >1 week after discontinuation of long-acting benzodiazepines used during detoxification. Subjects were excluded if they had positive urine drug screens or alcohol breathalyzer test on testing day. Positive cannabis urine drug screen was allowed for Cann subjects, because metabolites can be detected 3 weeks after last use.

The Meth subjects were tested 1 week–1 year after abstinence and excluded if they had current major depressive episode of moderate severity (Beck Depression Inventory [BDI] >20), other major psychiatric history, or medical illness. Because human immunodeficiency virus (HIV) frequency is high, a subanalysis was conducted. Other forms of substance addiction were allowed, assuming the primary drug for rehabilitation admission was methamphetamine (self-identified, highest frequency use, and escalating use before admission). Regular drug screens were conducted at the rehabilitation center. All psychiatric diagnoses were confirmed by a psychiatrist with DSM IV-TR criteria.

For HV, EtOH, obese BED, obese control subjects, and Cann subjects, two separate specifically designed questionnaires were used to assess drug use (e.g., type, duration of use, amount/week, last use). Psychiatric disorders were screened with the Mini International Neuropsychiatric Interview (10). Subjects completed the UPPS-P Impulsive Behaviour Scale (11) and BDI (12). The EtOH and obese subjects completed the Alcohol Use Disorders Identification Test (13), and obese subjects completed the Binge Eating Scale (BES) (14). The National Adult Reading Test (15) was used to obtain an index of premorbid IQ.

Subjects were paid for their study participation time and told they could receive an additional amount (£5) for their performance. Subjects in Minnesota were given the equivalent amount in a department store gift card. The study was approved by the University of Cambridge Research Ethics Committee and the University of Minnesota Institutional Review Board.

### Task

**Figure 1:**
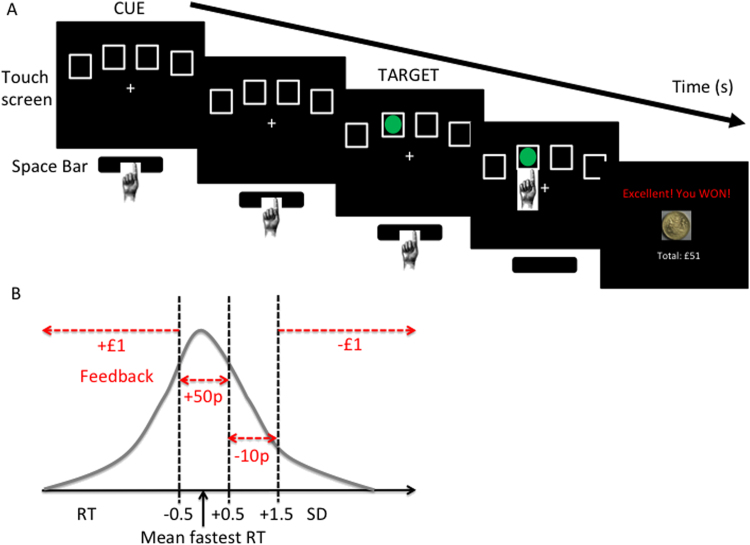
Premature responding task. **(A)** Task. Subjects press and hold down the space bar when they see four empty boxes (Cue) on the touch screen. After a green circle (Target) appears in one of the boxes, the subject releases the space bar and touches the box in which the target had appeared. The main outcome measure, premature responding, is measured as release of the space bar before target onset. **(B)** Feedback for the Test blocks is individualized on the basis of the mean fastest reaction time (RT) and SD obtained in the Baseline block.

Subjects were seated in front of a touch screen (a Paceblade Tablet personal computer; Paceblade Technology, Amersfoort, the Netherlands). When four boxes appeared on the screen, the subject pressed and held down the space bar on the keyboard with their dominant index finger (Figure 1). The space bar press indicated the “cue onset” time. After a specified period (cue-target interval), a green circle target appeared briefly and randomly in one of the four boxes. Subjects released the space bar and touched the box on the screen in which the target had appeared. The primary outcome measure was premature release of the space bar before target onset. The block order was as follows: Baseline block 1; Test block 1; Baseline block 2; Test blocks 2–4. Baseline blocks without monetary feedback were used to individualize monetary feedback amounts for subsequent blocks on the basis of the mean fastest reaction time (RT) and SD of the individual (Figure 1). The four Test blocks with monetary feedback were optimized to increase premature responding and varied by duration and variability of the cue-target interval and the presence of distractors. See Supplement 1 for further task details. It was programmed in Visual Basic with Visual Studio 2005 and Microsoft .NET Framework 2.0 (Microsoft, Redmond, Washington) with the US currency equivalent for feedback for subjects tested in Minnesota. Total task duration was 20 min.

Motivation to reward feedback was calculated as Motivation Index = (Mean RT Baseline 1 − Mean RT Baseline 2)/(Mean RT Baseline 1 + Mean RT Baseline 2). Test block 1 (with monetary feedback) occurred between Baseline blocks (without feedback) and had otherwise similar characteristics. Thus, Baseline 2 follows instrumental learning with monetary feedback consistent with testing in extinction without feedback. Other outcome measures included accuracy (correct responses/[correct responses + incorrect responses]), correct trial RT (in Test block 1), late responses (late responses/[correct fast responses + late responses]), which correspond to errors of omission in the rodent paradigm, and total won. Incorrect responses are errors of commission (wrong box touched after target onset) (1) and are equivalent to the accuracy measure: incorrect response = 1 − accuracy. Outlier RTs (RT >3 SD from mean) were removed from analysis.

The HV were tested on the stop signal task (16), a test of motor response inhibition and action cancellation, and a delay discounting task (17), which assesses the preference for a smaller immediate reward over a larger delayed reward. Primary outcome measures included the slope of the discounting curve (K-value), the go reaction time, and stop signal reaction time.

### Statistical Analysis

Variables that were not normally distributed (Shapiro-Wilk statistic *p* < .05) were transformed with square root transformation. Outliers (>3 SD above group mean) were removed. The EtOH, Cann, Obese BED, and Obese control subjects and Meth subjects were compared with their own matched HV with independent *t* tests. To control for differences in BDI or IQ, univariate analysis was conducted with BDI or IQ covariates. Relationships between premature responding and Motivation Index, disease severity measures, and task characteristics were tested with Pearson’s correlation coefficient *r*. Current smokers, ex-smokers, and nonsmokers were compared with analysis of variance. In Meth subjects, HIV+ and HIV− and high nicotine (>1 pack/day) and low or no nicotine were compared with independent *t* tests. Statistical tests were two-tailed, and significance was assigned at *p* < .05.

## Results

### Abstinent Alcohol-Dependent Subjects

Thirty EtOH subjects (reported in mean [SD]: weeks abstinent 15.60 [16.89]; years heavy use: 12.78 [8.27]; units/day: 28.36 [14.58]; Total units [units/day × years heavy use × 365 × percent drinking days]: 128,573 [124,490]) were compared with 30 HV. The EtOH subjects had higher UPPS-P, Alcohol Use Disorders Identification Test, and BDI scores (Table 1). The EtOH subjects were taking the following medications: acamprosate (*n* = 2); and disulfiram (*n* = 1).

**Table 1:** Subject Characteristics and Behavioral Measures

	EtOH	HV-EtOH			Cann	HV-Cann		
	(*n* = 30)	(*n* = 30)	*t*	*p*	(*n* = 30)	(*n* = 60)	*t*	*p*

Age	41.40 (11.57)	42.47 (12.35)	.35	.730	25.33 (7.53)	26.42 (7.74)	.64	.527
Men (*n*)	18	18			18	46		
IQ	114.32 (6.76)	116.13 (5.88)	1.11	.273	116.76 (5.89)	117.18 (5.70)	.32	.745
BDI	12.89 (9.29)	5.62 (6.47)	3.52	< .001	9.81 (8.99)	6.18 (6.73)	2.15	.034
UPPS-P	154.25 (20.14)	120.69 (26.29)	5.55	< .001	141.38 (19.52)	129.81 (22.03)	2.44	.017
AUDIT	19.59 (14.10)	5.15 (3.81)	5.42	< .001				
Premature Response	10.17 (8.79)	6.02 (4.36)	2.32	.024	10.39 (8.34)	7.01 (4.53)	2.50	.014
Accuracy	.93 (.05)	.92 (.06)	.70	.486	.89 (.09)	.91 (.07)	1.16	.250
Late Response	.06 (.05)	.08 (.07)	1.27	.208	.08 (.07)	.07 (.08)	.58	.562
Total Win	1087.53 (419.93)	1089.00 (400.10)	.01	.989	950.39 (413.33)	1044.06 (463.52)	.94	.352
RT Baseline 1	394.96 (97.69)	369.48 (112.48)	.94	.353	345.93 (113.77)	342.05 (97.97)	.17	.867
RT Reward	339.38 (50.76)	316.39 (48.35)	1.80	.077	319.82 (27.34)	304.15 (32.72)	.23	.831
RT Baseline 2	336.28 (91.83)	268.75 (63.89)	3.31	.002	258.99 (112.74)	256.90 (80.75)	.37	.714
Motivation Index	.08 (.10)	.16 (.12)	2.81	.007	.14 (.12)	.15 (.14)	.40	.679

Reported in mean (SD). AUDIT, Alcohol Use Disorders Identification Test; BDI, Beck Depression Inventory; BED, binge eating disorder; BES, Binge Eating Scale; BMI, body mass index; Cann, recreational cannabis users; EtOH, abstinent alcohol-dependent subjects; HV, healthy volunteers; RT, reaction time; UPPS-P, UPPS Impulsive Behaviour Scale.

**Figure 2:**
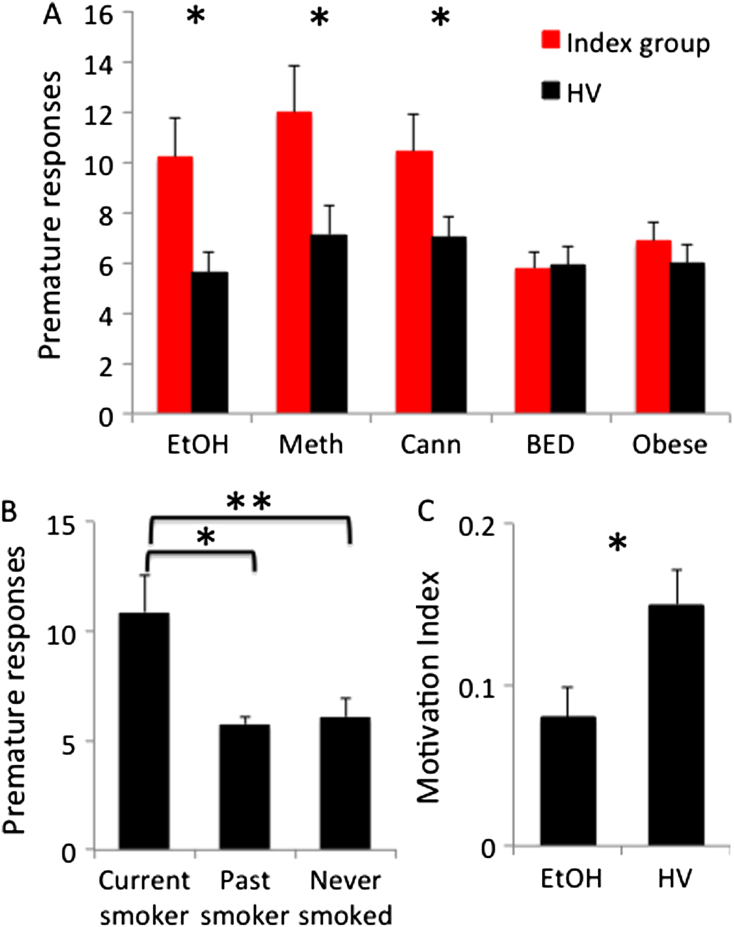
Premature responding and motivation index. **(A)** Premature responses in abstinent alcohol- (EtOH) (*n* = 30) and methamphetamine-dependent (Meth) (*n* = 23) subjects, recreational cannabis users (Cann) (*n* = 30), and obese subjects with binge eating disorder (BED) (*n* = 30) (Index group = red) versus healthy volunteers (HV) (black) and obese control subjects without BED (white) (*n* = 30). **(B)** Premature responses in current and past HV smokers and nonsmokers. **(C)** Motivation Index in EtOH (*n* = 30) versus HV. Error bars represent SEM. *p < .05; **p < .005.

Compared with HV, EtOH subjects made more premature responses (Figure 2, Table 1), including when covaried for BDI (*F* = 8.99, *p* = .004). The EtOH subjects also made more premature responses compared with HV (*t* = −2.36, *p* = .023) when subanalyzed to exclude the three subjects taking medications with possible psychotropic effects. In the secondary analysis, EtOH subjects had decreased motivation to reward feedback (Motivation Index) (Figure 2, Table 1). There were no differences in premature responding between current (*n* = 12, 10.71 [8.20]), past (*n* = 4, 9.5 [7.14]), and nonsmokers (*n* = 11, 10.72 [9.82]) (*F* = .06, *p* = .941).

In EtOH subjects, there was no correlation between premature responding and severity (total units), duration abstinence, or Motivation Index (reported as Pearson correlation coefficient: *r* = −.09–.18, *p* > .05). However, there was a negative correlation between Motivation Index with total units (*r* = −.43, *p* = .019).

### Recreational Cannabis Users

Thirty Cann subjects (reported in mean [SD]: number of joints/week 6.64 [5.21]; number of years 5.12 [4.21]; 5 did not report the amount) were compared with 60 HV. The Cann subjects had higher UPPS-P scores compared with HV. The Cann subjects made more premature responses compared with HV (Figure 2, Table 1), including when subanalyzed without those with positive cannabis urine drug screens (*n* = 19, premature responding 11.41 [8.13], *t* = 2.99, *p* = .004).

### Obese Subjects with and without BED

Thirty obese BED and 30 obese control subjects were compared with their own age- and gender-matched HV. The groups differed by BMI, BES, and BDI scores (Table 2). There were no differences in premature responses or Motivation Index between obese BED and HV and obese control subjects and HV (Figure 2, Table 2).

**Table 2:** Subject Characteristics and Behavioral Measures in Methamphetamine Dependence

	Obese BED	HV			Obese Control	HV			Meth	HV-Meth		
	(*n* = 30)	(*n* = 30)	*t*	*p*	(*n* = 30)	(*n* = 30)	*t*	*p*	(*n* = 22)	(*n* = 20)	*t*	*p*

Age	42.92 (8.59)	44.12 (10.18)	.49	.623	44.06 (9.70)	43.59 (10.01)	.18	.854	31.05 (4.78)	33.50 (7.78)	1.24	.221
Men (*n*)	13	13			19	19			21	16		
IQ	115.95 (6.67)	116.32 (5.93)	.23	.821	115.18 (6.45)	116.49 (5.89)	.82	.415	108.89 (4.59)	112.01 (4.71)	2.17	.036
BDI	12.50 (6.52)	5.02 (5.25)	4.89	< .001	6.96 (5.92)	5.93 (5.31)	.71	.481	15.32 (8.13)	5.35 (5.33)	4.74	< .001
UPPS-P	132.60 (19.98)	124.12 (23.53)	1.50	.138	128.95 (19.89)	123.95 (24.11)	.88	.384	156.71 (22.47)	120.94 (21.27)	5.22	< .001
BMI	34.68 (5.49)	23.86 (2.74)	9.66	< .001	32.72 (3.41)	24.11 (2.89)	10.55	< .001				
BES	24.70 (7.56)	7.22 (7.12)	9.22	< .001	8.67 (7.08)	7.30 (7.05)	.75	.456				
AUDIT	6.11 (5.51)	5.13 (3.78)	.80	.425	4.09 (3.99)	4.58 (3.87)	.48	.631				
Premature Responding	5.78 (3.66)	5.91 (4.18)	.13	.899	6.83 (4.73)	5.99 (4.35)	.72	.477	13.35 (6.77)	7.52 (5.59)	3.03	.004
Accuracy	.94 (.06)	.92 (.07)	1.19	.240	.94 (.04)	.92 (.07)	1.36	.180	.90 (.07)	.91 (.06)	.49	.624
Late Response	.08 (.07)	.08 (.08)	.00	1.000	.07 (.07)	.08 (.08)	.51	.608	.06 (.06)	.07 (.05)	.58	.563
Total Win	1126.79 (558.44)	1058.17 (418.35)	.54	.592	1189.21 (456.83)	1093.24 (433.28)	.83	.407	1009.15 (398.22)	1192.43 (418.29)	1.45	.154
RT Baseline 1	379.14 (96.54)	388.07 (103.01)	.35	.733	412.75 (93.27)	381.20 (99.35)	1.27	.210	348.53 (96.10)	389.24 (140.37)	1.11	.276
RT Reward	324.48 (40.82)	347.62 (36.09)	2.32	.021	315.84 (69.67)	335.49 (35.12)	1.38	.173	315.33 (43.24)	318.97 (36.83)	029	.772
RT Baseline 2	288.14 (86.55)	271.64 (64.89)	.84	.407	284.45 (66.40)	279.23 (65.12)	.31	.760	270.75 (121.25)	269.63 (94.95)	.03	.974
Motivation Index	.14 (.10)	.18 (.13)	1.36	.187	.19 (.14)	.18 (.13)	.29	.775	.12 (.13)	.18 (.09)	1.72	.09

Reported in mean (SD).

Meth, abstinent methamphetamine-dependent subjects; other abbreviations as in Table 1.

In obese subjects with and without BED, there was no correlation between premature responding and BES, BMI, or Motivation Index (*r* = −.19–.002, *p* > .05). There was a negative correlation between Motivation Index and BES (*r* = −.37, *p* = .012) but not BMI (*r* = .20, *p* = .128).

### Abstinent Methamphetamine-Dependent Subjects

Twenty-three Meth subjects (reported in mean [SD]: days abstinent: 79.16 [140.28]; years ever used: 10.16 [6.31]; years of heavy use: 2.60 [2.51]; Penn Craving Scale: 15.17 [9.17]) were compared with 20 HV from Minnesota (Table 2). Data from one Meth subject were excluded, due to a moderately severe current major depressive episode. Six Meth subjects had a concurrent alcohol use disorder, and 21 used nicotine daily. Meth subjects had the following comorbid psychiatric diagnoses: lifetime major depression (*n* = 4); panic disorder (*n* = 1); posttraumatic stress disorder (*n* = 1); obsessive-compulsive disorder (*n* = 1); anorexia nervosa/bulimia (*n* = 1); compulsive sexual behaviors (*n* = 2); and attention-deficit/hyperactivity disorder (*n* = 4). Meth subjects were taking the following medications: antidepressant (*n* = 9); mood stabilizer (*n* = 3) (used also for pain); neuroleptic (*n* = 2); and medication status unknown (*n* = 2).

The Meth subjects had a lower IQ and higher BDI and UPPS-P scores compared with HV (Table 2). The Meth subjects had higher premature responding compared with HV (Figure 2, Table 2) including when co-varied for BDI and IQ (*F* > 4, *p* < .05). There were no differences in premature responding (reported in mean [SD]: HIV+ 14.09 [11.32], HIV− 14.00 [6.29], *t* = .02, *p* = .981) or Motivation Index (HIV+ .12 [.14], HIV− .12 [.10], *t* = −.08, *p* = .947) in Meth subjects who were HIV− (*n* = 11) versus HIV+ (*n* = 11) or who had a concurrent alcohol use disorder (*n* = 6) versus those that did not (*n* = 16) (*t* = −1.75 –.21, *p* > .05). There were no differences in premature responding between heavy (>1 ppd, *n* = 11, 14.73 [7.72]) and light/nonsmokers (*n* = 9, 11.67 [5.36]) (*t* = 1.00, *p* = .332).

In Meth subjects, premature responding or Motivation Index did not correlate with duration of use, Penn Craving Scale score or duration of abstinence (*r* = −.33–.08, *p* = .141–.634).

### HV and Nicotine Use

Nicotine use was compared in a subgroup of HV. Current smokers (*n* = 19, 9 men [47%], age in years 30.00 [10.63], years smoked 12.27 [9.39]) had greater premature responding compared with past smokers (*n* = 12, 3 men [25%], age in years 39.75 [16.08], years smoked 6.31 [5.00]) and nonsmokers (*n* = 60, 28 men [47%], age in years 31.25 [11.89]) (age: *F* = 2.76, *p* = .07; gender: χ^2^ = 2.02, *p* = .357; premature responding: *F* = 4.51, *p* = .013) (Figure 2). Premature responding remained significantly different when covaried for age and gender (*F* = 4.22, *p* = .022).

### Relationship with Other Measures

In HV, premature responding did not correlate with the UPPS-P (reported as Pearson correlation coefficient *r*: *n* = 110, *r* = .13, *p* = .372), Barratt Impulsiveness Scale (*n* = 60, *r* = .14, *p* = .391), Motivation Index (*n* = 110, *r* = .09, *p* = .382), Go Reaction Time (mean 445.39 [SD 105.38]; *n* = 55, *r* = −.15, *p* = .327), stop signal reaction time (mean 175.71 [SD 45.11]; *n* = 55, *r* = .07, *p* = .62), or Delay Discounting Task (mean .02 [SD .02]; *n* = 80, *r* = .11, *p* = .397). In HV, premature responding was negatively correlated with age (*n* = 110, *r* = −.25, *p* = .004) but not IQ (*n* = 110, *r* = −.10, *p* = .50). When all groups were considered both in the HV and for each subject group, premature responding also did not correlate with Motivation Index (*r* = −.13–.16, *p* > .05) or with IQ, BDI, or task measures including accuracy, RT, late responses, or amount won (*p* > .05). There were no gender differences in HV or each subject group in premature responding or Motivation Index (*p* > .05).

## Discussion

We developed a novel task for premature responding adapted from the preclinical 5-CSRTT and demonstrate its translational utility in clinical subjects. In keeping with our hypotheses, the main findings indicate that subjects abstinent from alcohol and methamphetamine dependence exhibited significantly more premature responding compared with HV. Recreational cannabis users were also more impulsive on this novel task than HV, a behavioral impairment shared by current smokers but not ex-smokers or nonsmokers. However, obese subjects with and without binge eating did not show elevated levels of premature responding compared with HV. Obesity, with food as a natural reward, and substance addictions, with drug as an exogenous reward, have overlaps, but its relationship is not without controversy (18). The comparison of obesity and binge eating with substance addiction with the same measure allows a direct assessment of the relationship with substance addiction. That subjects with BED were not elevated in premature responding did not support our hypothesis. Here we highlight differences between obesity and binge eating with substance addictions, on the basis of the premature responding measure, a form of motor impulsivity. Our findings dovetail with recent preclinical studies showing premature responding is both a consequence of (i.e., a state effect) (5) and a predictor of and risk factor (i.e., a trait effect) for the transition to compulsive cocaine and nicotine use 19, 20. Because our study assesses subjects cross-sectionally after substance exposure, we are unable to distinguish between state and trait effects or to assess whether subjects are self-medicating a pre-existing condition. Future studies in unaffected family members are required to address these differences.

### Stimulant Dependence

In the stimulant dependence preclinical literature, rodents with high premature responding have lower ventral striatal D2/D3 receptors (21) and are at greater risk for the development of compulsive cocaine seeking. Thus, rodents with high levels of premorbid premature responding have greater motivation to take cocaine and inability to inhibit drug seeking, despite aversive consequences (8). In humans, low striatal D2/D3 receptor availability is associated with impulsivity in both methamphetamine-dependent subjects and HV, arguing for premorbid trait effects (22). Equally, in rodents, greater premature responding for up to 2 weeks after chronic methamphetamine exposure suggests premature responding can also be a consequence of methamphetamine abuse (5).

Methamphetamine blocks reuptake and enhances release of norepinephrine and dopamine and, to a much lesser extent, serotonin (23). In primates, methamphetamine is associated with striatal dopaminergic neurodegeneration 24, 25 with substantial but incomplete recovery after 18 months (26). In humans, persistently reduced dopamine transporter (27) and D2/D3 receptor density (28) is partially reversible after prolonged abstinence (27).

Several lines of preclinical evidence suggest that dopamine, serotonin, and norepinephrine modulate impulsive action. Rodents with high premature responding have lower ventral striatal D2/D3 receptors (21). Acute amphetamine increases premature responding in rodents, an effect attenuated by 6-hydroxydopamine lesions of the nucleus accumbens and by D1/D2 receptor antagonists (29). Central serotonin depletion or 5-HT2C receptor antagonism is also associated with greater premature responding in rodents (30). Atomoxetine, a selective norepinephrine-reuptake inhibitor also dose-dependently decreases premature responding (31). Studies of unaffected family members are indicated to differentiate possible neurochemical state and trait effects.

### Alcohol Dependence

Abstinent subjects with alcohol dependence also had greater premature responding. However, in contrast to the preclinical literature on stimulants, the role of premature responding as a predictor for alcohol dependence is less well-established. Premature responding was positively associated with greater withdrawal severity from chronic alcohol in a study of 15 different inbred strains of mice (32). Acute alcohol exposure (33) and early but not late abstinence after chronic alcohol exposure is associated with increased premature responding in rodents (6). Thus, premature responding is a state effect of alcohol, but its status as a trait effect is less clear.

### Binge Eating and Obesity

The relationship between premature responding and binge eating is also not well-established. In rodents, high premorbid premature responding is associated with greater escalation of sucrose-seeking behavior and reinstatement after extinction (34). By contrast, premature responding in 15 different strains of mice is not associated with sucrose acquisition or preference (32). Our finding that obese subjects were not elevated in premature responding is consistent with these preclinical findings.

### Cannabis Use

In rodents, the cannabinoid CB1 receptor antagonist, SLV330, decreases premature responding (7). Recreational cannabis users have elevated impulsivity as measured with questionnaires and behavioral tasks including impulsive choice, motor response inhibition, and reflection impulsivity 35, 36. In the present study, recreational cannabis users made more premature responses despite exclusion of those screening positive for cannabis metabolites, suggesting that our findings were not related to acute cannabis effects.

### Nicotine Use

Premature responding is associated with nicotine use as both a state and trait effect. In rodents, nicotine increases premature responding (37), and high premorbid premature responding predicts greater motivation to initiate and maintain nicotine use (20). That current smokers have greater premature responding, compared with ex-smokers and nonsmokers, suggests a clear state effect of nicotine that might be associated with greater likelihood of ongoing use but does not support a trait effect.

### Relationship to Other Tasks and Measures

We have stringently and operationally defined premature responding with a translation of the preclinical 5-CSRTT [itself based on a human paradigm; see Robbins (1)]. The task incorporates measures to optimize premature responding, including decreasing target time, variable cue-target intervals after repeated responding at fixed short intervals, and introduction of a distractor (1). The premature responding measure is also differentiated from other measures of inattention, accuracy, and sensitivity to reward feedback. Other studies have assessed impulsive action with continuous performance tasks in which subjects respond quickly to targets and must withhold responding to catch trials 38, 39. These tasks capture a form of motor impulsivity measured as commission errors consistent with motor response inhibition or action restraint assessed as in the Go/NoGo paradigm. Premature responding has also been assessed in the context of high conflict in the Simon task defined as rapid response errors (40) and rapid responding to high conflict stimuli (41). This form of premature responding might be more specific to situations of high conflict. Anticipatory responding has also been assessed in the context of risky time-sensitive rewards (42) and might also be sensitive to reward or loss value. Thus, other tasks measuring impulsive action might measure other forms of motor control or be specific to the context of conflict, risk, or reward sensitivity.

In this study, premature responding correlated negatively with increasing age, consistent with the trajectory of impulsivity with age (43). The measure did not correlate with impulsivity questionnaires, which is a common observation in the comparison of questionnaire and laboratory-based measures (44). We show that this novel task is independent of other subtypes of impulsivity, such as stopping and delay discounting. Inhibitory mechanisms might be implicated but might differ, depending on whether it is anticipatory or postinitiation. In the rodent 5-CSRTT, differences between premature responding and false errors (analogous to Go/NoGo or Continuous Performance Task commission errors) have been highlighted (45). The issue of proactive stopping (46) or preparing to suppress a response tendency rather than reactive stopping after signal onset might be relevant. In the rodent literature, premature responding can correlate with delay discounting (47), although the neural substrates might not be identical (4). That we did not observe a relationship might reflect task differences: in rodents, delay discounting is tested with short delays in seconds with rewarding feedback, whereas the questionnaire uses delays in days–months without feedback. A discounting task in real time with feedback is indicated (48). Premature responding did not correlate with IQ, depression scores, accuracy, RT, late responses, amount of money won, or motivation for monetary feedback. The accuracy measure accounts for variations in accuracy due to nonspecific influences such as attentional capacity, motivation, or motor behavior, because correct and incorrect responses require the same motor effort (1).

### Motivation

We also show that motivation for monetary incentive as measured by the Motivation Index is decreased in abstinent alcohol-dependent subjects and is negatively correlated with severity of alcohol dependence and binge eating. This measure of motivation, which assesses RT in extinction after instrumental conditioning with monetary feedback, is unrelated to premature responding. Because monetary reward in these disorders is a conditioned reinforcer, our findings suggest possible similarities between substance use disorders and a subtype of obesity characterized by the pattern of food intake or binge eating. This finding dovetails with the observation of decreased ventral striatal activity to anticipation of monetary reward with an increase in activity to alcohol cues in alcohol-dependent subjects (49). These data are thus consistent with rodent studies in which sugar bingeing demonstrates addictive-like properties including enhanced responding for sugar after abstinence, amphetamine cross-sensitization, and nucleus accumbens dopamine release (50). In humans, food presentation to BED subjects is associated with greater striatal dopamine release (51). Our data add to the growing literature addressing the relationship between obesity and substance use disorders.

### Study Limitations

There were several limitations. In the obese subjects, testing under food restriction might influence these findings. Using the primary reinforcer (e.g., food) might also affect these findings, although monetary outcome is a conditioned reinforcer in all the diagnostic groups tested. Nicotine use was not defined a priori as a group, and the sample size of ex-smokers was low although well-matched for age and gender. Further studies focusing on nicotine use are indicated, although these preliminary findings are strongly suggestive of a possible effect. Relative to HV, methamphetamine-dependent subjects were not matched for gender and had a lower IQ, and methamphetamine and alcohol-dependent subjects had higher depression scores. However, we show that IQ, gender, and depression scores are unrelated to these measures, suggesting these factors to be relatively unimportant. Other forms of impulsivity have also been shown to be independent of IQ (52). Lower premorbid IQ has been reported to be associated with stimulant dependence (53). Cognitive changes might also change with more prolonged abstinence.

### Conclusions

With a novel translational task, we show that premature responding is elevated in subjects with substance dependence and recreational cannabis use but not in obese subjects with or without binge eating. Alcohol use and binge eating severity were linked by a blunted motivation for monetary rewards. Our findings help to elucidate the complex relationship between drug and natural food reward and suggest binge eating might represent a specific subtype in the mechanisms underlying obesity. Studies in high-risk populations are warranted to assess the role of premature responding as a biomarker for the development of substance addiction.
