# Supplementary material for: Measuring “Waiting” Impulsivity in Substance Addictions and Binge Eating Disorder in a Novel Analogue of Rodent Serial Reaction Time Task
Source: Biol Psychiatry. 2014 Jan 15;75(2):148–55. doi: 10.1016/j.biopsych.2013.05.013 (PMC3988873; doi:10.1016/j.biopsych.2013.05.013)
Supplement: Supplementary file 1 — Supplementary Material [file mmc1.pdf]

Measuring 'Waiting' Impulsivity in Substance Addictions and Binge Eating Disorder in a Novel Analogue of the Rodent Serial Reaction Time Task

Supplemental Information

Task Description

The premature responding task consists of two Baseline blocks and four Test blocks.

Baseline Blocks: The Baseline blocks were used to calculate the individual's mean reaction time (RT) and standard deviation (SD) in order to individualize feedback according to the individual's RT and encourage individuals to respond faster. As RT can vary across the trial for many reasons including experience with the task or fatigue, the task included 2 Baseline blocks. The first Baseline block occurred at the start of the trial with the mean RT used for Test Block 1. The second Baseline block occurred at the end of Test Block 1 with the mean RT from both Baseline blocks used for Test blocks 2 to 4. The subjects were told to respond as quickly as possible during the Baseline blocks and the words 'Keep going' appeared on the screen as feedback.

Test Block Feedback (Figure 1B): Each Baseline block had 20 trials with the final 10 trials used to calculate mean RT and SD to individualize feedback and incentivize faster responding in subsequent Test blocks. On Test blocks subjects saw both feedback (text and corresponding monetary image) and the cumulative total. The relationship between Baseline block mean RT, SD and Test block feedback were as follows:

<u>Very fast accurate responses</u>: For very fast accurate responses in which RT during a trial in the Test blocks was less than -0.5 SD of the Baseline RT, the response was followed by the text "YOU WIN!! EXCELLENT!!" along with a £1 image. If subjects won £1 in three sequential trials, the feedback increased to £2.

<u>Fast accurate responses</u>: For accurate responses in which Test RT was between -0.5 SD and +0.5 SD of the Baseline RT, the response was followed by the text "Very good. Keep going."

along with a 50 pence image. Test RTs that were accurate and between +0.5 SD and +1.5 SD of the Baseline RT were followed by the text “Good. Keep going.” along with a 10 pence image.

Slow accurate responses: Slow but accurate responses in which trial RTs were greater than +1.5 SD of the Baseline RT were penalized and followed by the text “YOU LOSE!! TOO LATE!! HURRY UP!!” and an image of -£1 with a red X over the coin.

No response: If no responses were registered, the feedback was “TOO LATE!! GO FASTER!!” with an image -£1 with a red X.

Premature response or incorrect responses: Neither premature responses (responding prior to target onset) nor incorrect responses (touching the incorrect box) were penalized. Following a premature response, subjects were required to touch the screen to complete the trial, which was followed by the text ‘Keep going’. An incorrect response was followed by the text ‘Keep going’.

Test Blocks: There were 4 Test blocks with monetary feedback (40 trials per block). Subjects were instructed to respond as quickly as possible. They were told that they would earn money for their responses and would earn more money for faster responses. They were told that it was more important to be fast rather than accurate and that they would not lose money if they were inaccurate.

In the Baseline blocks without feedback, the target duration was 64 msec and the cue-target interval was 2 seconds. In Test Block 1 (Long Target) with monetary feedback, the target duration and cue-target interval were the same as the Baseline blocks. In Test Block 2 (Short Target), the target duration was 32 msec and the cue-target interval was 2 seconds. In Test Block 3 (Variable Interval), the target duration was 32 msec and the cue-target interval varied from 2 to 10 seconds. In Test Block 4 (Distractor), red circles followed by yellow circles appeared sequentially and randomly in one of the four boxes during the cue-target

interval (2 to 10 seconds) prior to onset of the green target (target duration 32 msec). The distractor circles were presented for 32 msec for a random number. The distance between the touch screen and keyboard was held constant for each individual throughout the course of the experiment.
